# Comparison of surgical complications after curative surgery in patients with oral cavity squamous cell carcinoma and sarcopenia

**DOI:** 10.1002/jcsm.13162

**Published:** 2022-12-23

**Authors:** Shuang Zeng, Chia‐Hao Chang, Mingyang Sun, Wan‐Ming Chen, Szu‐Yuan Wu, Jiaqiang Zhang

**Affiliations:** ^1^ Department of Anesthesiology and Perioperative Medicine, Henan Provincial People's Hospital People's Hospital of Zhengzhou University Zhengzhou Henan China; ^2^ Academy of Medical Sciences of Zhengzhou University Zhengzhou Henan China; ^3^ Department of Otorhinolaryngology Lo‐Hsu Medical Foundation, Lotung Poh‐Ai Hospital Luodong Taiwan; ^4^ Graduate Institute of Business Administration, College of Management Fu Jen Catholic University Taipei Taiwan; ^5^ Artificial Intelligence Development Center Fu Jen Catholic University Taipei Taiwan; ^6^ Department of Food Nutrition and Health Biotechnology, College of Medical and Health Science Asia University Taichung Taiwan; ^7^ Big Data Center Lo‐Hsu Medical Foundation, Lotung Poh‐Ai Hospital Luodong Yilan Taiwan; ^8^ Division of Radiation Oncology Lo‐Hsu Medical Foundation, Lotung Poh‐Ai Hospital Luodong Taiwan; ^9^ Department of Healthcare Administration, College of Medical and Health Science Asia University Taichung Taiwan; ^10^ Cancer Center Lo‐Hsu Medical Foundation, Lotung Poh‐Ai Hospital Luodong Taiwan; ^11^ Centers for Regional Anesthesia and Pain Medicine, Taipei Municipal Wan Fang Hospital Taipei Medical University Taipei Taiwan; ^12^ Department of Management, College of Management Fo Guang University Jiaoxi Taiwan

**Keywords:** Sarcopenia, OCSCC, Perioperative, Postoperative, Adverse outcomes

## Abstract

**Background:**

The study aims to clarify the association of sarcopenia with perioperative and postoperative complications in oral cavity squamous cell carcinoma (OCSCC) patients undergoing curative surgery and to understand the reasons causing the poor oncologic outcomes for OCSCC.

**Methods:**

We conducted a propensity score matching study to investigate the association of perioperative and postoperative outcomes in OCSCC patients with sarcopenia and without sarcopenia. A retrospective analysis of a large national data set from the Taiwan Cancer Registry Database was conducted. At least two claims for patients with a principal diagnosis of sarcopenia within the 12‐month preoperative period were defined as the criteria for sarcopenia diagnosis (ICD‐10‐CM code M62.84). Sarcopenia was diagnosed through the measurement of low muscle strength and low muscle mass by any one of the patient's attending orthopaedic physician, rehabilitation physician, family medicine specialist or geriatrician. A multivariate logistic regression model was used to calculate the perioperative, and postoperative major complications.

**Results:**

Our final cohort included 16 293 patients with OCSCC (10 862 and 5 431 in the sarcopenia and nonsarcopenia groups, respectively) who were eligible for further analysis. The sarcopenia group was 10.40% female and 89.60% male, and the nonsarcopenia group was 9.74% female and 90.26% male. The mean age ± standard deviation (*SD*) were 56.44 ± 11.14 and 56.22 ± 11.29 for sarcopenia and nonsarcopenia groups. OCSCC patients with sarcopenia undergoing curative surgery had a significantly higher blood transfusion rate and volume; longer intensive care unit (ICU) stay, and hospital stay; higher postoperative 30‐day mortality (adjusted odds ratio [aOR]: 1.12, 95% confidence interval [CI] [1.07, 1.56]) and rates of pneumonia (aOR: 1.34, 95% CI [1.20, 1.50]), acute renal failure (aOR: 1.45, 95% CI [1.12, 1.87]) and septicaemia (aOR: 1.29, 95% CI [1.15, 1.45]); higher postoperative first‐year mortality (aOR: 1.18, 95% CI [1.13, 1.51]) and rates of pneumonia (aOR: 1.43, 95% CI [1.30, 1.56]), acute myocardial infarction (aOR: 1.52, 95% CI [1.06, 2.18]) and septicaemia (aOR: 1.29, 95% CI [1.15, 1.45]).

**Conclusions:**

OCSCC patients with sarcopenia might exhibit more perioperative and surgical complications than those without sarcopenia.

## Introduction

Head and neck cancer is the third most common cancer in Taiwan and the fifth leading cause of cancer death in men.^1^ Oral cavity squamous cell carcinoma (OCSCC) accounts for more than 80% of head and neck cancer cases in Taiwan, whereas in Western countries, oropharyngeal cancer accounts for most head and neck cancer cases.^1^ In addition, according to the latest Global Cancer Alliance (IARC, WHO) 2020 report, 377 713 new cases of oral cavity cancer and 177 757 deaths occur worldwide each year.^2^ Curative surgery is the primary treatment for OCSCC according to the National Comprehensive Cancer Network (NCCN) guidelines.^1^ Therefore, understanding the perioperative and postoperative complications is crucial to improve surgical outcomes in patients with OCSCC.

Sarcopenia is an independent risk factor for mortality, cancer recurrence, and distant metastasis in patients with OCSCC undergoing curative surgery,^3^ but the underlying reasons remain unclear. Sarcopenia also increases the risk of postoperative complications.^4^ Therefore, the association of sarcopenia with perioperative and postoperative complications might contribute to mortality, locoregional recurrence and distant metastasis in OCSCC patients with sarcopenia, which has raised our interest. If the association between sarcopenia and adverse surgical outcomes in OCSCC could be determined, the prevention, early detection and treatment of preoperative sarcopenia would be important in the susceptible population scheduled for OCSCC surgery^5^ and improve their survival and surgical outcomes.

We conducted a head‐to‐head propensity score matching (PSM) study involving patients with OCSCC with and without sarcopenia to clarify the association of sarcopenia with perioperative and postoperative complications among patients with OCSCC undergoing curative surgery and to explore the reasons underlying the poor oncologic outcomes of OCSCC.

## Patients and methods

### Data sources

We enrolled patients with OCSCC who underwent curative surgery—tumour resection and neck dissection—between 1 January 2016, and 31 December 2018; their data were obtained from the Taiwan Cancer Registry Database (TCRD). The follow‐up period was from the index date (i.e., the date of surgery) to 31 December 2020. The types of neck dissection and their indications were supraomohyoid neck dissection for clinically N0 tumours,^6^ modified neck dissection for ipsilateral clinically positive nodes^7^ and bilateral neck dissection for contralateral metastases or tumours that crossed the midline.^8^ Adjuvant treatments were administered according to the NCCN guidelines and the patients' tolerance.^9^ The TCRD contains detailed cancer‐related patient data, including the clinical cancer stage, cigarette smoking habits, treatment modalities, pathological data and the grade of differentiation.^1^ The study protocols were reviewed and approved by the Institutional Review Board of Tzu‐Chi Medical Foundation (IRB109‐015‐B).

### Inclusion and exclusion criteria

The diagnoses of the enrolled patients were confirmed after reviewing their pathological data. All patients with OCSCC underwent curative‐intent surgery. The inclusion criteria were as follows: Patients who were aged ≥20 years had a new diagnosis of pathologic Stage I–IVB OCSCC without metastasis (determined in accordance with the American Joint Committee on Cancer criteria [AJCC] 7th edition), had no other cancer and underwent tumour resection and neck dissection. Patients were excluded if they had a history of other cancers before the index date, had an unknown pathological stage, had missing sex data, had an unclear differentiation of tumour grade or had a nonsquamous cell carcinoma pathologic type.

In October 2016, the US Centers for Disease Control and Prevention formally recognized sarcopenia as a disease and coded it as M62.84 in ICD‐10‐CM.^10^ We defined sarcopenia according to the ICD‐10‐CM code M62.84 after 2016.^11^ At least two claims for patients with a principal diagnosis of sarcopenia within the 12‐month preoperative period were defined as the criteria for sarcopenia diagnosis. In Taiwan, sarcopenia was defined as the skeletal muscle mass index (SMI) of 2 standard deviations (*SD*s) or more below the normal sex‐specific means for young persons.^12^ Sarcopenia was diagnosed through the measurement of low muscle strength and low muscle mass by any one of the patient's attending orthopaedic physician, rehabilitation physician, family medicine specialist or geriatrician.

Sarcopenia diagnosis was determined based on the presence of at least two claims within the 12‐month preoperative period in patients with the principal diagnosis of sarcopenia. The enrolled patients were divided into two groups depending on whether or not they had sarcopenia before their OCSCC diagnosis (the sarcopenia group and the nonsarcopenia group). Patients given a diagnosis of sarcopenia after surgery were excluded. Patients receiving induction chemotherapy before surgery were excluded in the study because induction chemotherapy would be a bias for the surgical complications, and there have been weak evidences for induction chemotherapy in OCSCC. Moreover, synchronous second primary were excluded in the current study.

### PSM and covariates

To reduce the effects of potential confounders when comparing postoperative adverse outcomes between the sarcopenia and nonsarcopenia groups, all patients were matched through PSM using the following variables: age, sex, OCSCC sites, AJCC pathologic stage, pT, pN, differentiation, surgical margin, lymphovascular invasion, adjuvant treatments, CCI scores, current smoking, excessive alcohol consumption, income levels, urbanization and medical centre or not (Supporting information, *Table* *S1*). We matched the cohorts at a ratio of 1:2 by using the greedy matching method, and covariates were matched with a propensity score within a calliper of 0.2.^13^ Comorbidities were determined according to the *International Classification of Diseases, Ninth Revision, Clinical Modification* (ICD‐9‐CM) and were defined as the main diagnosis in inpatient records or if the number of outpatient visits was ≥2 within 1 year. Comorbidities that presented 6 months before the index date were recorded. Repeat comorbidities were excluded from CCI scores to prevent repetitive adjustment in multivariate analysis. After adjustments for confounders, a multivariate logistic regression model was used to examine 30‐day, first‐year, second‐year and third‐year postoperative major complications from the index date (surgical date) in patients with and without preoperative sarcopenia.

In this study, continuous variables are presented as means ± *SD*s, where appropriate. A 1:2 PSM applied here for the sarcopenia and nonsarcopenia groups is a common technique used for selecting controls with identical background covariates as those of study participants to minimize differences among the study patients (that we deem necessary to be controlled based on the previous studies).^14^ A multivariate logistic regression model was performed to analyse postoperative major complications and related variables in the sarcopenia and nonsarcopenia groups.^15^ Multivariate logistic regression analysis was performed to calculate odd ratios (ORs) with 95% confidence intervals (CIs) for determining whether preoperative sarcopenia was the potential independent predictor for postoperative 30‐day, first‐year, second‐year and third‐year major complications.

### Outcome measures

Eight major postoperative major complications were monitored: acute myocardial infarction, acute renal failure, pneumonia caused by deep‐wound infection, postoperative bleeding, pulmonary embolism, septicaemia and stroke.^14^ These complications and subsequent overall in‐hospital mortality within 30 days after the index surgery were the primary outcomes of the current study.^14^ The perioperative outcomes of the amount and rate of blood transfusion (mL) and length of hospital stay (days) were the dependent variable. Events recorded within the first, second and third postoperative years are considered chronic complications because sarcopenia is a long‐term chronic disease contributing to chronic surgical oncological complications.^16^ Instead of 30‐day mortality and postoperative major complications, the first‐year, second‐year and third‐year postoperative mortality and complications are a valid measure of surgical quality, especially oncologic surgery, but these measures are unclear in OCSCC surgery.^16^ Therefore, major complications in the first, second and third postoperative years were considered the secondary outcomes in our study.

### Data analysis

We used the *χ* ^2^ test to analyse the descriptive parameters for demographic status and coexisting medical conditions and compare postoperative major complications and death rates between surgical patients with and without sarcopenia. Continuous variables were analysed using *t* tests to compare differences between the sarcopenia and nonsarcopenia groups. Two types of multivariate mixed models accounting for patient clusters in hospitals were used to ascertain the association of sarcopenia with the outcomes: general linear regression models including continuous outcomes; the amount of blood transfused; and hospital stay, with propensity score adjustment for age, sex, OCSCC sites, AJCC pathologic stage, pT, pN, differentiation, surgical margin, lymphovascular invasion, adjuvant treatments, CCI scores, current smoking, excessive alcohol consumption, income levels, urbanization and medical centre or not; and logistic regression models including postoperative outcomes and surgical outcomes, with propensity score adjustment for the same aforementioned variables. SAS v.9.4 (SAS Institute, Cary, NC, USA) was used for all statistical analyses; differences between groups were considered significant if two‐sided *P* values were <0.05.

## Results

### Study cohort

Our final cohort included 16 293 patients with OCSCC (10 862 and 5431 in the sarcopenia and nonsarcopenia groups, respectively) who were eligible for further analysis; their characteristics are listed in *Table* *S1*. Furthermore, after PSM, the between‐group differences in age, sex, OCSCC sites, AJCC pathologic stage, pT, pN, differentiation, surgical margin, lymphovascular invasion, adjuvant treatments, CCI scores, current smoking, excessive alcohol consumption, income levels, urbanization and medical centre or not were not significant.

### Perioperative outcomes: blood transfusion volume and rate and hospital stay days stratified by sarcopenia

Significant differences were observed in the blood transfusion volume and mean blood transfusion volume between the sarcopenia and nonsarcopenia groups (44.17% vs. 39.60% and 421.70 mL vs. 354.75 mL, both *P* < 0.0001, Table 1). The sarcopenia group had longer intensive care unit (ICU) stay and hospital stay (median: 1.50 days and 11 days, respectively) than the nonsarcopenia group (median: 1.00 day and 7 days, respectively, *P* < 0.0001, *Table* *1*).

**TABLE 1:** General linear regression analysis of perioperative blood transfusion volume and length of hospital stay propensity score–matched OCSCC patients with or without sarcopenia

	Nonsarcopenia		Sarcopenia		
	*N* = 10 862		*N* = 5431		*P* value^a^
Perioperative complications	*N*	%	*N*	%	
Blood transfusion incidence	10 862		5431		<0.0001
No	6561	60.40%	3032	55.83%	
Yes	4301	39.60%	2399	44.17%	
Blood transfusion volume (mL, mean ± *SD*)	354.75 ± 882.48		421.70 ± 1074.07		<0.0001
Blood transfusion volume (median, IQR)	220.00 (0.00, 500.00)		255.00 (0.00, 500.00)		<0.0001
Postoperative admission					
ICU stay (days, mean ± SD)	0.67 ± 0.85		0.75 ± 0.91		<0.0001
ICU stay days; median (IQR, Q1, Q3)	1.00 (0.00, 1.00)		1.50 (0.00, 1.00)		<0.0001
Hospital stay (days, mean ± SD)	7.11 ± 17.93		13.84 ± 21.33		<0.0001
Hospital stay, days; median (IQR)	7.00 (0.00, 10.00)		11.00 (0.00, 17.00)		<0.0001

Abbreviations: ICU, intensive care unit; IQR, interquartile range; *N*, numbers; OCSCC, oral cavity squamous cell carcinoma; *SD*, standard deviation.

^a^ General linear regression for continuous variables with propensity score adjustment of age, sex, OCSCC sites, AJCC pathologic stage, pT, pN, differentiation, surgical margin, lymphovascular invasion, adjuvant treatments, CCI scores, current smoking, excessively consuming alcohol, income levels, urbanization and medical centre or not.

### Adjusted odds ratio and 95% confidence intervals for 30‐day postoperative adverse outcomes

After adjustment for age, sex, OCSCC sites, AJCC pathologic stage, pT, pN, differentiation, surgical margin, lymphovascular invasion, adjuvant treatments, CCI scores, current smoking, excessive alcohol consumption, income levels, urbanization, and medical centre or not, multivariate logistic regression analyses revealed that the sarcopenia group had a significantly higher risk of 30‐day postoperative mortality (adjusted odds ratio [aOR]: 1.12, 95% confidence interval [CI] [1.07, 1.56]) and 30‐day major complications, including postoperative pneumonia (aOR: 1.34, 95% CI [1.20, 1.50]), postoperative acute renal failure (aOR: 1.45, 95% CI [1.12, 1.87]), and septicaemia (aOR: 1.29, 95% CI [1.15, 1.45]) (*Table* *2*).

**TABLE 2:** Adjusted ORs and 95% CIs for 30‐day postoperative outcomes associated with propensity score–matched OCSCC patients with or without sarcopenia undergoing curative surgery

	Nonsarcopenia		Sarcopenia		*P* value	OR	*P* value	aOR^a^	*P* value
	*N* = 10 862		*N* = 5431						
	*N*	%	*N*	%					
30‐day mortality	50	0.46%	45	0.83%	0.0446	1.24 (1.08, 1.52)	0.0052	1.12 (1.07, 1.56)	0.0016
Pneumonia	948	8.73%	605	11.14%	<0.0001	1.311 (1.18, 1.46)	<.0001	1.34 (1.20, 1.50)	<0.0001
Acute myocardial infarction	71	0.65%	36	0.66%	0.9453	1.014 (0.68, 1.52)	0.9452	1.04 (0.7, 1.56)	0.8416
Acute renal failure	142	1.31%	102	1.88%	0.0047	1.445 (1.12, 1.87)	0.0049	1.45 (1.12, 1.87)	0.0050
Deep‐wound infection	23	0.21%	16	0.29%	0.3076	1.393 (0.74, 2.64)	0.3094	1.37 (0.72, 2.61)	0.3339
30‐day postoperative bleeding	228	2.10%	116	2.14%	0.8775	1.018 (0.81, 1.28)	0.8772	1.02 (0.82, 1.28)	0.8475
Pulmonary embolism	6	0.06%	6	0.11%	0.2205	2.001 (0.65, 6.21)	0.2297	2.06 (0.66, 6.42)	0.2115
Septicaemia	848	7.81%	524	9.65%	<.0001	1.261 (1.13, 1.41)	<.0001	1.29 (1.15, 1.45)	<0.0001
Stroke	449	4.13%	230	4.23%	0.7604	1.026 (0.87, 1.21)	0.7595	1.03 (0.87, 1.21)	0.7237

Abbreviations: aOR, adjusted odd ratio; CI, confidence interval; *N*, numbers; OCSCC, oral cavity squamous cell carcinoma; OR, odd ratio.

^a^ Logistic regression for binary variables with propensity score adjustment of age, sex, OCSCC sites, AJCC pathologic stage, pT, pN, differentiation, surgical margin, lymphovascular invasion, adjuvant treatments, CCI scores, current smoking, excessively consuming alcohol, income levels, urbanization and medical centre or not.

### Adjusted ORs and 95% CIs for chronic surgical complications

The sarcopenia group had a significantly higher risk of first‐year mortality (aOR: 1.18, 95% CI [1.13, 1.51]) and first‐year major complications, including postoperative pneumonia (aOR: 1.43, 95% CI [1.30, 1.56]), acute myocardial infarction (aOR: 1.52, 95% CI [1.06, 2.18]), and septicaemia (aOR: 1.29, 95% CI [1.15, 1.45]) (*Table* *3*).

**TABLE 3:** Adjusted ORs and 95% CIs for first postoperative year outcomes associated with propensity score–matched OCSCC patients with or without sarcopenia undergoing curative surgery

	Nonsarcopenia		Sarcopenia		*P* value	OR	*P* value	aOR^a^	*P* value
	*N* = 10 862		*N* = 5431						
	*N*	%	*N*	%					
First‐year mortality	2081	19.16%	1359	25.02%	0.0002	1.24 (1.18, 1.52)	0.0024	1.18 (1.13, 1.51)	0.0003
Pneumonia	1490	13.72%	964	17.75%	<.0001	1.381 (1.26, 1.51)	<0.0001	1.43 (1.30, 1.56)	<0.0001
Acute myocardial infarction	71	0.65%	52	0.96%	0.0347	1.487 (1.04, 2.13)	0.0304	1.52 (1.06, 2.18)	0.0234
Acute renal failure	197	1.81%	118	2.17%	0.1166	1.217 (0.97, 1.53)	0.0948	1.23 (0.98, 1.55)	0.0789
Deep‐wound infection	23	0.21%	16	0.29%	0.3076	1.393 (0.74, 2.64)	0.3094	1.37 (0.72, 2.61)	0.3339
Postoperative bleeding	228	2.10%	116	2.14%	0.8775	1.018 (0.81, 1.28)	0.8772	1.02 (0.82, 1.28)	0.8475
Pulmonary embolism	6	0.06%	6	0.11%	0.2205	2.001 (0.65, 6.21)	0.2297	2.06 (0.66, 6.42)	0.2115
Septicaemia	848	7.81%	524	9.65%	<.0001	1.261 (1.13, 1.41)	<.0001	1.29 (1.15, 1.45)	<0.0001
Stroke	449	4.13%	230	4.23%	0.7604	1.026 (0.87, 1.21)	0.7595	1.03 (0.87, 1.21)	0.7237

Abbreviations: aOR, adjusted odd ratio; CI, confidence interval; *N*, numbers; OCSCC, oral cavity squamous cell carcinoma; OR, odd ratio.

^a^ Logistic regression for binary variables with propensity score adjustment of age, sex, OCSCC sites, AJCC pathologic stage, pT, pN, differentiation, surgical margin, lymphovascular invasion, adjuvant treatments, CCI scores, current smoking, excessively consuming alcohol, income levels, urbanization and medical centre or not.

OCSCC patients with sarcopenia undergoing curative surgery exhibited increased odds of mortality in the second year compared with those without sarcopenia (aOR: 1.07, 95% CI [1.03, 1.34]; *Table* *4*). In the second year after curative surgery, OCSCC patients with sarcopenia exhibited increased odds of pneumonia compared with those without sarcopenia (aOR: 1.27, 95% CI [1.14, 1.42]) as well as significantly increased septicaemia and stroke risks compared with those without sarcopenia (aOR: 1.86, 95% CI [1.15, 4.62] and aOR: 1.20; 95% CI [1.04, 1.38] for septicaemia and stroke, respectively).

**TABLE 4:** Adjusted ORs and 95% CIs for second postoperative year outcomes associated with propensity score–matched OCSCC patients with or without sarcopenia undergoing curative surgery

	Nonsarcopenia		Sarcopenia		*P* value	OR	*P* value	aOR^a^	p value
	*N* = 8781		*N* = 4072						
	*N*	%	*N*	%					
Second‐year mortality	1460	16.63%	793	19.47%	0.0435	1.15 (1.04, 1.4)	0.0113	1.07 (1.03, 1.44)	0.0031
Pneumonia	1110	12.64%	618	15.18%	<.0001	1.237 (1.11, 1.38)	<.0001	1.27 (1.14, 1.42)	<0.0001
Acute myocardial infarction	69	0.79%	40	0.98%	0.2583	1.253 (0.85, 1.85)	0.2591	1.28 (0.87, 1.9)	0.2148
Acute renal failure	127	1.45%	63	1.55%	0.6594	1.071 (0.79, 1.45)	0.6594	1.09 (0.8, 1.48)	0.5855
Deep‐wound infection	40	0.46%	17	0.42%	0.7627	0.916 (0.52, 1.62)	0.7627	0.92 (0.52, 1.62)	0.7595
Wound bleeding	30	0.34%	18	0.44%	0.3853	1.098 (0.95, 1.27)	0.2065	1.13 (0.97, 1.32)	0.1094
Pulmonary embolism	10	0.11%	9	0.22%	0.1413	1.295 (0.72, 2.33)	0.3864	1.29 (0.71, 2.32)	0.4058
Septicaemia	517	5.89%	331	8.13%	0.0001	1.94 (1.29, 4.79)	0.0087	1.86 (1.15, 4.62)	0.0012
Stroke	484	5.51%	245	6.02%	0.2497	1.171 (1.02, 1.35)	0.0263	1.20 (1.04, 1.38)	0.0126

Abbreviations: aOR, adjusted odd ratio; CI, confidence interval; *N*, numbers; OCSCC, oral cavity squamous cell carcinoma; OR, odd ratio.

^a^ Logistic regression for binary variables with propensity score adjustment of age, sex, OCSCC sites, AJCC pathologic stage, pT, pN, differentiation, surgical margin, lymphovascular invasion, adjuvant treatments, CCI scores, current smoking, excessively consuming alcohol, income levels, urbanization, and medical centre or not.

In the third year, the odds of chronic surgical complications, most postoperative major complications and mortality (aOR: 1.07, 95% CI [1.03, 1.30]) were not significantly increased in the sarcopenia group compared with the nonsarcopenia group. However, compared with the nonsarcopenia group, the sarcopenia group had increased risks of pneumonia (aOR: 1.46, 95% CI [1.27, 1.67]), septicaemia (aOR: 1.34, 95% CI [1.11, 1.61]) and stroke (aOR: 1.24, 95% CI [1.04, 1.47]).

## Discussion

Sarcopenia is associated with increased disability and decreased survival.^17^ It is an independent prognostic factor for poor survival in patients with head and neck cancer undergoing surgical radiotherapy or concurrent chemoradiotherapy.^18^ OCSCC patients with sarcopenia have poor oncologic outcomes, and those with head and neck cancer and sarcopenia have higher mortality, although the underlying reasons remain unclear.^3^ This study is the first PSM study with the largest sample size so far to thoroughly evaluate not only perioperative complications (e.g., blood transfusion, ICU stay and hospital stay) but also acute and chronic postoperative complications in patients with OCSCC with or without sarcopenia undergoing curative surgery. To the best of our knowledge, this is the first study to demonstrate that OCSCC patients with sarcopenia undergoing curative surgery had a significantly higher blood transfusion rate and volume; longer ICU stay and hospital stay; higher postoperative 30‐day mortality and pneumonia, acute renal failure and septicaemia rates; higher first‐year postoperative mortality and pneumonia, acute myocardial infarction and septicaemia rates; higher second‐year postoperative mortality and pneumonia, septicaemia and stroke rates; and higher third‐year postoperative mortality and pneumonia, septicaemia and stroke rates than those without sarcopenia.

Studies and systematic reviews have extensively demonstrated that individuals with sarcopenia are at risk of functional decline, frailty, decreased mobility, falls, fractures and hospitalization, all of which can increase mortality risk.^19^ Sarcopenia is associated with a significantly higher risk of mortality, independent of the population, sarcopenia definition, follow‐up period and risk of bias. In an umbrella review that included 30 meta‐analyses of sarcopenia and adverse outcomes, sarcopenia was significantly associated with poorer prognosis across 12 cancer types: gastric, hepatocellular, urothelial, head and neck, haematologic, pancreatic, breast, colorectal, lung, oesophageal and ovarian cancer.^20^ All the studies included in the umbrella review indicated the consistent trend that sarcopenia was associated with poor survival in patients with cancer (all hazard ratios: 1.11–2.12).^20^ Sarcopenia increases the risk of recurrence and cancer‐related mortality in gastric, urothelial, colorectal and hepatocellular cancer.^20^ The highest hazard ratios of mortality were noted in gastric, pancreatic, hepatocellular and head and neck cancer.^20^ Similarly, in a recent meta‐analysis of sarcopenia in malignant haematologic diseases, sarcopenia was an independent predictor of mortality (hazard ratio, 1.94).^21^ Five meta‐analyses examining sarcopenia and 16 postoperative outcomes in gastrointestinal, oesophageal, gastric or colorectal cancer concluded that sarcopenia was significantly related to one or more of major postoperative complications in each cancer; however, these studies have not included data for head and neck cancers.^22^ In addition, there is existing research for negative outcomes associated with sarcopenia in OCSCC, but none/little for pre‐existing sarcopenia (sarcopenia diagnosed before OCSCC). Sarcopenia was associated most strongly with postoperative pulmonary complications, infections, readmission rates and length of hospitalization (hazard ratios: 1.35–6.24) in these studies.^22^ However, no real‐world large‐sample‐size study was conducted to examine the impact of sarcopenia in patients with OCSCC undergoing curative surgery; ours is the first study to demonstrate that OCSCC patients with sarcopenia undergoing curative surgery had more perioperative and acute postoperative complications than those without sarcopenia (*Tables* *1*, *2*, *3*, *4*, *5*), which is consistent with the results of the aforementioned studies.^20^, ^22^

**TABLE 5:** Adjusted ORs and 95% CIs for third postoperative year outcomes associated with propensity score–matched OCSCC patients with or without sarcopenia undergoing curative surgery

	Nonsarcopenia		Sarcopenia		*P* value	OR	*P* value	aOR^a^	*P* value
	*N* = 7231		*N* = 3279						
	*N*	%	*N*	%					
Third‐year mortality	1477	20.43%	756	23.06%	<.0001	1.14 (1.07, 1.37)	<.0001	1.07 (1.03, 1.30)	<.0001
Pneumonia	632	8.74%	396	12.08%	<.0001	1.434 (1.26, 1.64)	<.0001	1.46 (1.27, 1.67)	<0.0001
Acute myocardial infarction	60	0.83%	30	0.91%	0.6607	1.104 (0.71, 1.71)	0.6608	1.11 (0.71, 1.73)	0.6479
Acute renal failure	72	1.00%	47	1.43%	0.0494	1.446 (1, 2.09)	0.0507	1.45 (1.00, 2.10)	0.0517
Deep‐wound infection	35	0.48%	18	0.55%	0.6633	1.135 (0.64, 2.01)	0.6635	1.15 (0.65, 2.03)	0.6427
Wound bleeding	12	0.17%	6	0.18%	0.8449	1.103 (0.41, 2.94)	0.845	1.04 (0.38, 2.87)	0.9353
Pulmonary embolism	5	0.07%	5	0.15%	0.1992	2.207 (0.64, 7.63)	0.2109	2.30 (0.65, 8.13)	0.1947
Septicaemia	332	4.59%	196	5.98%	0.0026	1.321 (1.1, 1.58)	0.0026	1.34 (1.11, 1.61)	0.0019
Stroke	395	5.46%	214	6.53%	0.0306	1.208 (1.02, 1.44)	0.0308	1.24 (1.04, 1.47)	0.0170

Abbreviations: aOR, adjusted odd ratio; CI, confidence interval; *N*, numbers; OCSCC, oral cavity squamous cell carcinoma; OR, odd ratio.

^a^ Logistic regression for binary variables with propensity score adjustment of age, sex, OCSCC sites, AJCC pathologic stage, pT, pN, differentiation, surgical margin, lymphovascular invasion, adjuvant treatments, CCI scores, current smoking, excessively consuming alcohol, income levels, urbanization and medical centre or not.

Onoue et al. discovered that heart failure event‐free survival rates (including heart failure‐related hospitalization and deaths) were significantly lower in the sarcopenia group^23^; this is in agreement with our finding that the rate of acute myocardial infarction in the first postoperative year was higher in the sarcopenia group than in the nonsarcopenia group. Dillon et al. evaluated 92 patients with oral cancer after surgery and reported that the most sensitive predictors of cardiovascular complications were operating room time and estimated blood loss.^24^ The findings are compatible with our result that the sarcopenia group had higher blood transfusion volume and rate, leading to a higher incidence of acute myocardial infarction.^24^ The higher rate of cardiovascular complications in the sarcopenia group might be attributed to a higher chronic stroke risk in the sarcopenia group compared with that in the nonsarcopenia group (*Tables* *4* *and* *5*), because acute myocardial infarction has been considered a cause for ischemic stroke.^25^ In addition, the higher 30‐day postoperative acute renal failure might be attributed to the higher blood transfusion volume and rate in the sarcopenia group resulting from more blood loss.^26^

The 30‐day, first‐, second‐ and third‐year postoperative pneumonia rates were significantly higher in the sarcopenia group than in the nonsarcopenia group. Because the mass and strength of skeletal muscles are affected by many factors such as age, sex, underlying diseases, dietary habits and exercise, they can reflect the overall functional status of the body.^27^ In general, the prognosis of surgical patients is also closely related to the functional status of the body.^28^ Sarcopenia patients, as a special group, often exhibit low physical function, and they may have an increased risk of postsurgical complications.^29^ However, sarcopenia will lead to the reduction of skeletal muscle mass and the weakening of respiratory and swallowing muscles, resulting in atelectasis, pneumonia, dysphagia and malnutrition in patients.^30^ These aforementioned factors might increase postoperative pneumonia, prolong ICU or hospital stay, affect quality of life, and increase healthcare costs and mortality.^4^ Therefore, surgeons and anaesthesiologists should pay special attention to OCSCC patients with sarcopenia, as a potential high‐risk group for postoperative adverse outcomes. Our study is the leading study to show that the acute and chronic postoperative pneumonia risks are significantly higher in OCSCC patients with sarcopenia undergoing curative surgery.

In the current study, sarcopenia was the independent risk factor for 30‐day, first‐, second‐ and third‐year postoperative septicaemia in patients with OCSCC undergoing curative surgery. Although our study is the first to show a high septicaemia rate in OCSCC patients with sarcopenia undergoing curative surgery, the association of sarcopenia with postoperative infection and delayed recovery from colorectal cancer resection surgery has been reported.^31^ In addition, sarcopenia was an independent preoperative predictor of infection after pancreaticoduodenectomy.^32^ Although the mechanism by which sarcopenia is a risk factor for postoperative infections remains unclear, muscle mass depletion seems to be a risk factor for perioperative infection.^33^ Skeletal muscle is a secretory organ in which cytokines and other peptides are produced and released by muscle fibres,^34^ and adipose tissue is considered a key component of the immune system.^33^ Therefore, skeletal muscle depletion with an increase in adipose tissue leads to the synthesis and secretion of several proinflammatory cytokines and adipocytokines.^35^ Among them, increased adipokines and decreased interleukin‐15 levels might be related to the interaction between sarcopenia and immune senescence.^36^

Our results provide valuable information on the comprehensive postoperative care problems in OCSCC patients with sarcopenia undergoing curative surgery, especially regarding perioperative blood loss, postoperative mortality, pneumonia, acute renal failure, acute myocardial infarction, septicaemia and stroke. Establishing a comprehensive protocol to prevent or at least minimize the risks of the aforementioned perioperative and postoperative complications will reduce surgical mortality and complications in patients with OCSCC, especially given that sarcopenia is a potentially avoidable and reversible condition.^5^ Sarcopenia prevention might improve the survival of and reduce postoperative complications in the population at risk of developing OCSCC, such as those with excessive alcohol use, smoking or betel nut chewing.^37^ Measures to prevent or alleviate sarcopenia include increased strength exercise; adequate supplementation with proteins, vitamin D, antioxidants, long‐chain polyunsaturated fatty acids and other nutrients^38^; and muscle growth inhibitor antibodies.^39^ Short‐term resistance exercise training and protein supplementation before surgery can increase the mass and strength of skeletal muscles and reduce fat content in patients, thereby improving their immunity and reducing the incidence of postoperative pulmonary complications.^5^ Therefore, preoperative sarcopenia, swallowing function and respiratory muscle strength should be improved before elective surgery in patients with cancer to reduce the incidence of postoperative pneumonia.

This study has several limitations. First, all patients were enrolled from an Asian population in Taiwan. Second, although we used PSM to balance known confounders to reduce bias, unexpected confounders might exist for perioperative or postoperative complications.^13^ Third, the effective interventions for the sarcopenia might be the confounding factors in the current study, although the intervals of sarcopenia newly diagnosis to surgical date were similar (the mean ± *SD* periods of sarcopenia newly diagnosis to cancer surgery were 0.94 ± 0.34 years) during the follow‐up period. The majority of effective interventions for the sarcopenia were 3 months of interventions needed^40^; therefore, the interference of the effective interventions for the sarcopenia might be small. Moreover, because of the presence of effective interventions for the sarcopenia in the sarcopenia group, the perioperative and surgical complications might be underestimated in this group. Thus, our study findings regarding OCSCC patients with sarcopenia having more perioperative and surgical complications than those without sarcopenia would not be overturned. Fourth, some diseases included in the CCI could be directly or indirectly linked to sarcopenia and perioperative or postoperative complications; thus, adjustment of comorbidities and CCI would be necessary, although the root cause of sarcopenia was unavailable in the current study. Additionally, this study did not include information on dietary habits and body mass index, all of which may be risk factors for perioperative or postoperative complications.

The strengths of this study are its large sample size and the long‐term follow‐up of chronic postoperative complications. To the best of our knowledge, this is the leading, largest and long‐term follow‐up cohort study to examine the perioperative, acute and chronic postoperative complications between OCSCC patients with and without sarcopenia. According to our findings, sarcopenia was associated with more perioperative, acute and chronic postoperative complications in men with OCSCC. These findings should be further investigated in future clinical practice and prospective clinical trials.

## Conclusions

The medical resource consumption in the nonsarcopenia group was less in terms of the blood transfusion volume and rate, ICU stay and hospital stay compared with the sarcopenia group. OCSCC patients with sarcopenia may exhibit higher short‐term to long‐term surgical complications (postoperative mortality, pneumonia, acute renal failure, acute myocardial infarction, stroke and septicaemia) than those without sarcopenia.

## Funding

Lo‐Hsu Medical Foundation, LotungPoh‐Ai Hospital, supports Szu‐Yuan Wu's work (funding numbers: 11001, 11010, 11013 and 11103).

## Conflict of interest

The authors have no potential conflicts of interest to declare. The datasets supporting the study conclusions are included within the manuscript.

## Supporting information


**Table S1.** Clinicodemographic characteristics of OCSCC patients with or without sarcopenia
