# Supplementary material for: Comparison of surgical complications after curative surgery in patients with oral cavity squamous cell carcinoma and sarcopenia
Source: J Cachexia Sarcopenia Muscle. 2022 Dec 23;14(1):576–84. doi: 10.1002/jcsm.13162 (PMC9891945; doi:10.1002/jcsm.13162)
Supplement: Supplementary file 1 — Table S1. Clinicodemographic characteristics of OCSCC patients with or without sarcopenia [file JCSM-14-576-s001.docx]

**Supplemental TABLE 1.** Clinicodemographic characteristics of OCSCC patients with or without sarcopenia

|  | **Nonsarcopenia** | | **Sarcopenia** | | **p value** |
| --- | --- | --- | --- | --- | --- |
|  | **N = 10,862** | | **N = 5,431** | |  |
|  | **N** | **%** | **N** | **%** |  |
| **Age** (mean ± SD) | 56.22 ± 11.29 | | 56.44 ± 11.14 | | 0.2323 |
| Age, median (IQR, Q1, Q3), years old | 56.00 (49.00,63.00) | | 56.00 (49.00,63.00) | | 0.2988 |
| Age Groups (years old) |  |  |  |  | 0.9263 |
| <50 | 2,966 | 27.31% | 1,490 | 27.44% |  |
| 50–59 | 3,912 | 36.02% | 1,966 | 36.20% |  |
| ≥60 | 3,984 | 36.68% | 1,975 | 36.37% |  |
| **Sex** |  |  |  |  | 0.1829 |
| Male | 9,804 | 90.26% | 4,866 | 89.60% |  |
| Female | 1,058 | 9.74% | 565 | 10.40% |  |
| **Years of diagnosis** |  |  |  |  | 0.4577 |
| 2007–2010 | 2,208 | 20.33% | 1,148 | 21.14% |  |
| 2011–2014 | 4,569 | 42.06% | 2,248 | 41.39% |  |
| 2015–2018 | 4,085 | 37.61% | 2,035 | 37.47% |  |
| **OCSCC sites** |  |  |  |  | 0.1845 |
| Buccal | 8,145 | 74.99% | 4,011 | 73.85% |  |
| Gingival and mouth floor | 2,572 | 23.68% | 1,334 | 24.56% |  |
| Oral tongue | 145 | 1.33% | 86 | 1.58% |  |
| **AJCC pathologic stage** |  |  |  |  | 0.2362 |
| I | 2,514 | 17.70% | 1192 | 16.61% |  |
| II | 1,583 | 11.14% | 817 | 11.40% |  |
| III | 1229 | 8.65% | 681 | 9.50% |  |
| IVA | 5003 | 35.21% | 2471 | 34.45% |  |
| IVB | 533 | 3.73% | 270 | 3.76% |  |
| **pT** |  |  |  |  | 0.2378 |
| pT1 | 2,457 | 22.41% | 1,231 | 22.43% |  |
| pT2 | 2,527 | 23.05% | 1,258 | 22.92% |  |
| pT3 | 2,467 | 22.50% | 1,389 | 25.58% |  |
| pT4a | 2,960 | 27.25% | 1,350 | 24.86% |  |
| pT4b | 451 | 4.15% | 203 | 3.73% |  |
| **pN** |  |  |  |  | 0.7823 |
| pN0 | 5256 | 48.39% | 2608 | 48.02% |  |
| pN1 | 1,513 | 13.93% | 756 | 13.92% |  |
| pN2 | 3971 | 36.56% | 1985 | 36.55% |  |
| pN3 | 122 | 1.12% | 82 | 1.51 |  |
| **Differentiation** |  |  |  |  | 0.2281 |
| I (well differentiated) | 2689 | 24.76% | 1341 | 24.69% |  |
| II (moderately differentiated) | 6745 | 59.61% | 3237 | 59.60% |  |
| III | 1428 | 13.15% | 853 | 15.71% |  |
| **Surgical margin** |  |  |  |  | 0.5958 |
| Margin clear | 9514 | 87.59% | 4725 | 87.00% |  |
| Margin positive | 1,348 | 12.41% | 706 | 13.00% |  |
| **Lymphovascular invasion** |  |  | 3803 |  | 0.9550 |
| No | 4930 | 45.39% | 2480 | 45.66% |  |
| Yes | 5932 | 54.61% | 2,066 | 54.34% |  |
| **Adjuvant treatment** |  |  |  |  | 0.7213 |
| Adjuvant CCRT | 3,758 | 34.60% | 2,313 | 42.59% |  |
| Adjuvant chemotherapy | 307 | 2.83% | 163 | 3.00% |  |
| Adjuvant sequential chemotherapy and RT | 1,838 | 16.92% | 1,099 | 20.24% |  |
| Adjuvant RT | 1,915 | 17.63% | 776 | 14.29% |  |
| No adjuvant | 3,044 | 28.02% | 1,080 | 19.89% |  |
| **Adjuvant RT, Total Dose (Gy)** (mean ± SD) | 63.89 ± 16.05 | | 63.78 ± 15.35 | | 0.8131 |
| median (IQR, Q1–Q3) | 66.00 (60.00–70.00) | | 66.00 (60.00–70.00) | | 0.1348 |
| **Adjuvant cumulative platinum dose** (mg/m^2^) (mean ± SD) | 552.27 ± 431.55 | | 540.59 ± 413.19 | | 0.1831 |
| median (IQR, Q1–Q3) | 450.00 (300.00–680.0 | | 450.00 (300.00–650.0) | | 0.4228 |
| **CCI Scores** |  |  |  |  |  |
| Mean (mean ± SD) | 0.70 ± 1.11 | | 0.73 ± 1.13 | | 0.1395 |
| Median (IQR, Q1–Q3) | 0.00 (0.00–1.00) | | 0.00 (0.00–1.00) | | 0.2016 |
| **CCI Scores** |  |  |  |  | 0.2732 |
| 0 | 6,979 | 64.25% | 3,442 | 63.38% |  |
| ≥1 | 3,883 | 35.75% | 1,989 | 36.62% |  |
| **Current smoking** | 6735 | 62.00% | 3400 | 62.60% | 0.8629 |
| **Excessively consuming alcohol** | 1,488 | 13.70% | 744 | 13.70% | 0.9993 |
| **Income levels (NTD)** | 92 | 0.85% | 55 | 1.01% | 0.5262 |
| Low income | 6,806 | 62.66% | 3,328 | 61.28% |  |
| ≤20000 | 1,857 | 17.10% | 950 | 17.49% |  |
| 20001–30000 | 1,442 | 13.28% | 758 | 13.96% |  |
| 30001–45000 | 665 | 6.12% | 340 | 6.26% |  |
| >45000 | 92 | 0.85% | 55 | 1.01% |  |
| **Urbanization** |  |  |  |  | 0.8249 |
| Rural | 3,749 | 34.51% | 1,884 | 34.69% |  |
| Urban | 7,113 | 65.49% | 3,547 | 65.31% |  |
| **Medical center** |  |  |  |  | 0.8795 |
| No | 2,814 | 25.91% | 1,413 | 26.02% |  |
| Yes | 8,048 | 74.09% | 4,018 | 73.98% |  |

RT, radiotherapy; CCRT, concurrent chemoradiotherapy; CCI, Charlson comorbidity index; SD, standard deviation; IQR, interquartile range; AJCC, American Joint Committee on Cancer; N, numbers; Gy, Gray; pT, pathologic tumor stages; pN, pathologic nodal stages; OCSCC, oral cavity squamous cell carcinoma; NTD, New Taiwan Dollars.

.
